# Development of an immune-related prognostic index associated with hepatocellular carcinoma

**DOI:** 10.18632/aging.102926

**Published:** 2020-03-19

**Authors:** Bo Hu, Xiao-Bo Yang, Xin-Ting Sang

**Affiliations:** 1 Department of Liver Surgery, Peking Union Medical College Hospital, Chinese Academy of Medical Sciences and Peking Union Medical College, Beijing 100010, China

**Keywords:** hepatocellular carcinoma, oncogenes, immune system, prognosis, precision medicine

## Abstract

Liver hepatocellular carcinoma (LIHC), an inflammation-associated cancer induced by a variety of etiological factors, is still one of the most prevalent and lethal cancers in human population. In this study, the expression profiles of immune-related genes (IRGs) were integrated with the overall survival (OS) of 378 LIHC patients based on the Cancer Genome Atlas (TCGA) dataset. Moreover, the differentially expressed and survival related IRGs among LIHC patients were predicted through the computational difference algorithm and COX regression analysis. As a result, 7 genes, including HSPA4, S100A10, FABP6, CACYBP, HDAC1, FCGR2B and SHC1, were retrieved to construct a predictive model associated with the overall survival (OS) of LIHC patients. Typically, the as-constructed model performed moderately in predicting prognosis, which was also correlated with tumor grade. Functional enrichment analysis revealed that the genes of high-risk group were actively involved in mRNA binding and the spliceosome pathway. Intriguingly, the prognostic index established based on IRGs reflected infiltration by multiple types of immunocytes. Our findings screen several IRGs with clinical significance, reveal the drivers of immune repertoire, and illustrate the importance of a personalized, IRG-based immune signature in LIHC recognition, surveillance, and prognosis prediction.

## INTRODUCTION

Liver hepatocellular carcinoma (LIHC) is the sixth most prevalent cancer and third leading cause of cancer-related deaths worldwide [1]. It can be managed by surgical treatment and chemotherapy, but the mortality rate remains high [2]. For patients with advanced LIHC, chemotherapy fails to demonstrate a survival benefit [3, 4]. By contrast, therapeutic regimens that counteract the immunosuppressive mechanisms have the potential to dramatically alter the clinical outcomes of LIHC, which lead us to further explore the relationship of the abnormal immune gene expression with LIHC development and prognosis [5]. Cancer immunotherapy is a primary driver of personalized medicine, which makes aggressive efforts to leverage the immune system against tumors and is thus promising for treating human diseases [6, 7]. As an inflammation-associated tumor, the immunosuppressive microenvironment of LIHC is well evidenced to promote immune tolerance and evasion through various mechanisms, rendering LIHC an attractive candidate for immunotherapy [8]. Recently, immune checkpoint inhibitors (ICIs), including ipilimumab (the CTLA4 inhibitor) and nivolumab (the PD-1 inhibitor), have demonstrated great survival benefits for LIHC [9, 10]. According to a phase I/II study on nivolumab among patients with advanced LIHC, a 19% response rate (including a 5% complete response rate) is achieved, which is expected to be further increased by the combined therapies with different ICIs or the combined treatment of small molecules with ICIs [10]. However, it remains largely unknown about the molecular features of immune checkpoints and their correlations with clinicopathological parameters and LIHC tumor microenvironment (TME). Moreover, the molecular mechanisms of immunology in LIHC, especially for the immunogenomic effects, are still unclear so far. With the establishment and completion of the large-scale gene expression datasets, cancer researchers are able to identify the responsible biomarkers for tumor monitoring and surveillance in a rapid and accurate way [11–13]. For instance, Yang et al. proposed an immune-related classifier for the prognosis of cutaneous melanoma, which was derived from immune-related genes (IRGs) and demonstrated a powerful predictive ability [14]. Nevertheless, the clinical relevance and prognostic significance of IRGs in LIHC have to be explored.

This study aimed to gain insight into the potential clinical utility of IRGs in prognosis stratification, as well as their implicational potential as biomarkers for targeted LIHC therapy. We are committed to construct a robust immune-related signature to improve prognostic prediction of LIHC via comprehensive genomic data analysis. Results obtained in this study can provide certain foundation for subsequent in-depth immune-related studies, which show great promise for treating LIHC with personalized medicine.

## RESULTS

### Identification of the differentially expressed IRGs

**Figure 1:**
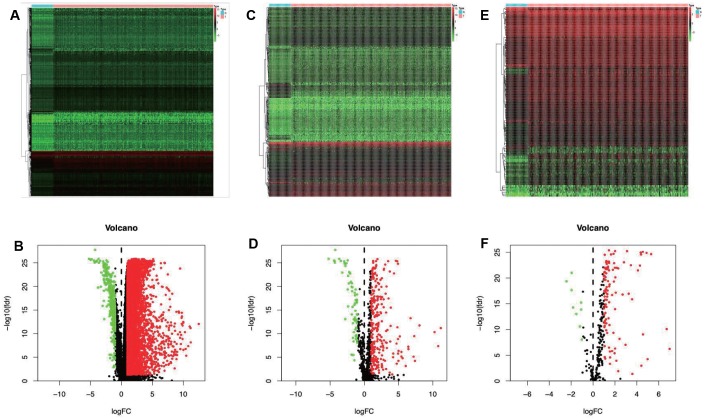
**Differentially expressed immune-related genes and transcription factors (TFs).** Heatmap (**A**) and volcano plot (**C**) demonstrating differentially expressed genes between hepatocellular carcinoma (LIHC) and non-tumor tissues. Differentially expressed immune-related genes (IRGs) are shown in heatmap (**B**) and volcano plot (**D**), red and green dots represent differentially expressed genes. Heatmap (**E**) and volcano plot (**F**) illustrating differentially TFs between LIHC and non-tumor tissues, red dots represent differentially up-regulated TFs. Red dots represent differentially up-regulated expressed genes, green dots represent differentially down-regulated expressed genes and black dots represent no differentially expressed genes. N, normal tissue. T, tumor.

A total of 7667 differentially expressed genes (DEGs) were identified using the edgeR algorithm, including 7273 up-regulated and 394 down-regulated ones (Figure 1A and 1B). Then, altogether 329 differentially expressed IRGs were extracted from these 7667 DEGs, including 267 up-regulated and 62 down-regulated ones (Figure 1C and 1D).

### Identification of the survival-related IRGs and construction of TF regulatory network

**Figure 2:**
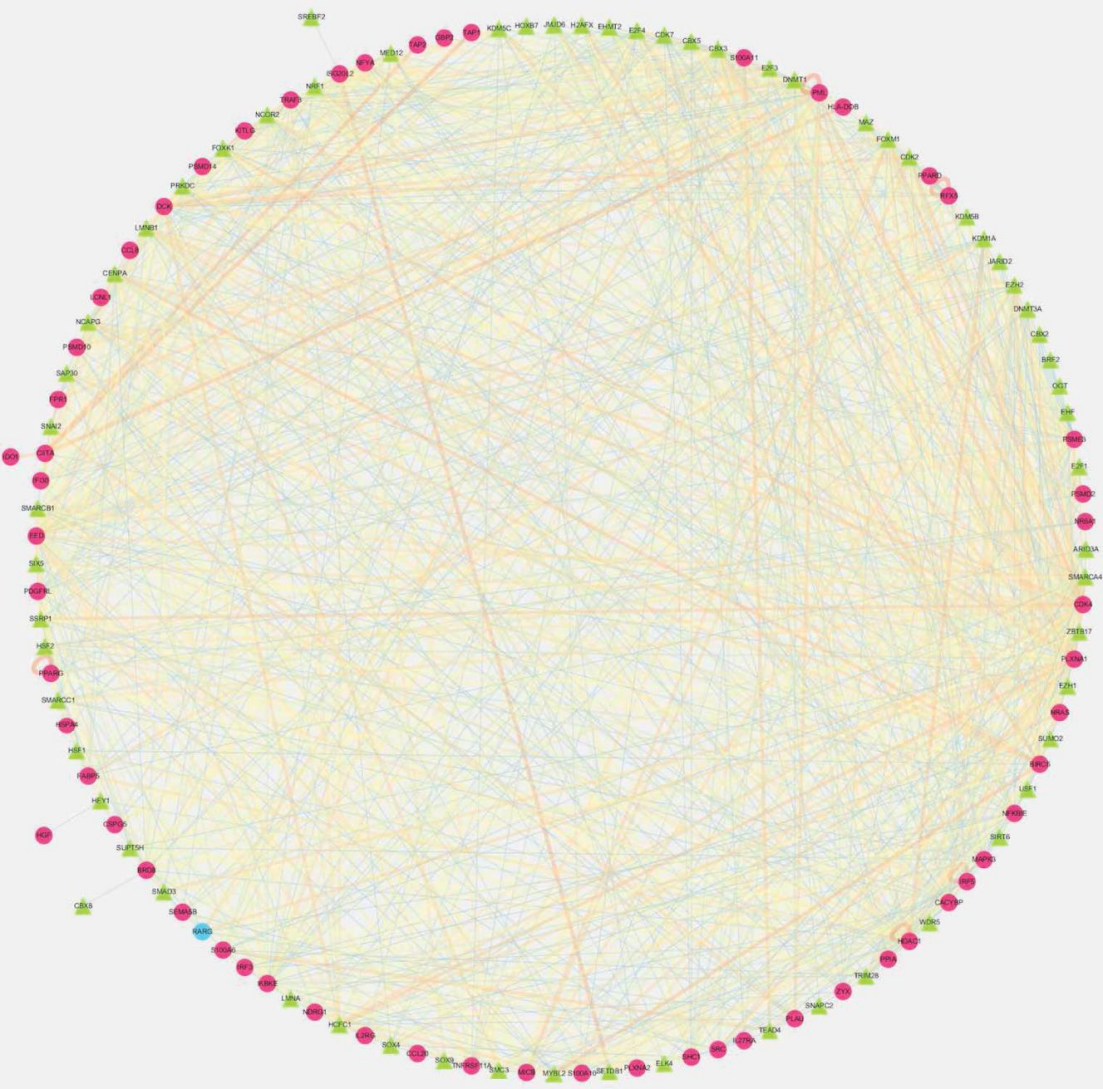
**Transcription factors (TFs)- mediated regulatory network.** Regulatory network constructed based on survival relevant TFs and IRGs. The red circle represents high-risk genes, the blue circle represents low-risk genes. The green triangle represents transcription factors, and the thickness and brightness of lines between nodes represent the level of relevance (low Cor values to small sizes and dark colors).

After screening, 61 IRGs were identified to show remarkable correlation with OS for TCGA-LIHC patients (P<0.01) (Supplementary Table 1). To explore the potential molecular mechanisms corresponding to the clinical significance of our survival-related IRGs, the regulatory mechanisms of these genes were also investigated. Specifically, the expression patterns of 318 TFs were examined, which suggested that 116 of them were differentially expressed between TCGA-LIHC and non-tumor hepatic samples (Figure 1E and 1F). Afterwards, the associations of these differentially expressed TFs with the prognostic immune genes were also analyzed. Finally, 64 factors were screened to construct the regulatory networks with 58 survival-related immune genes, as selected at the correlation score of > 0.4 and the P-value of <0.001. Notably, the TF-based regulatory schematic acutely illustrated the regulatory relationships among these IRGs (Figure 2).

### Development and validation of the immune-related prognostic signature in TCGA

**Figure 3:**
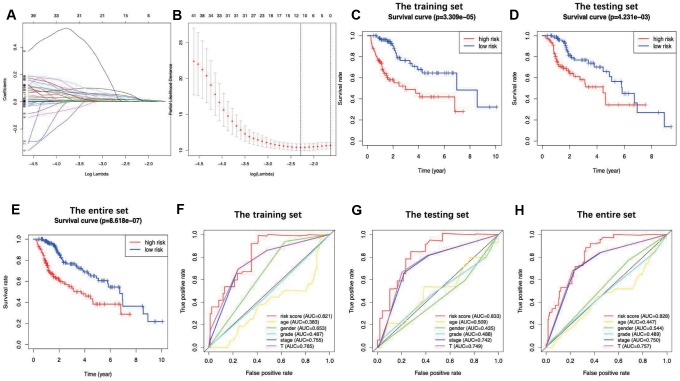
LASSO coefficient profiles of the 7 immune genes are depicted in (**A**, **B**) show the selection of the tuning parameter (lambda) in the LASSO model by tenfold cross-validation based on minimum criteria for OS; the lower X axis shows log (lambda), and the upper X axis shows the average number of OS-genes. The Y axis indicates partial likelihood deviance error. Red dots represent average partial likelihood deviances for every model with a given lambda, and vertical bars indicate the upper and lower values of the partial likelihood deviance errors. The vertical black dotted lines define the optimal values of lambda, which provides the best fit. Survival curves of patients in high risk group and low risk group of the training set (**C**), the testing set (**D**) and the entire set (**E**) are shown. Patients in high-risk group suffered shorter overall survival. (**F**–**H**) show survival-dependent receiver operating characteristic (ROC) curves validation at 1 – year of prognostic value of the prognostic index in the three sets (the training set, the testing set and the entire set, respectively).

**Figure 4:**
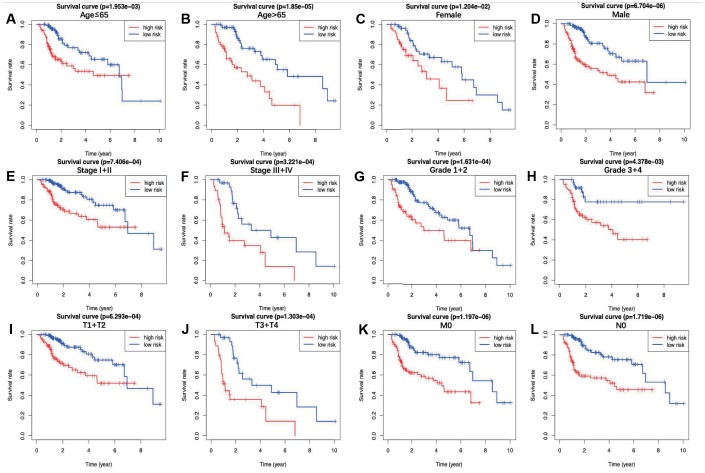
The overall survival differences between the high-risk group and the low-risk group were shown under the conditions of classifying patients by age (**A**, **B**), sex (**C**, **D**), stage (**E**, **F**), grade (**G**, **H**) and T stage (**I**, **J**). Patients without distant metastasis (**K**) and lymph node metastasis (**L**) are also displayed. Detailed notes are described in the main text.

**Figure 5:**
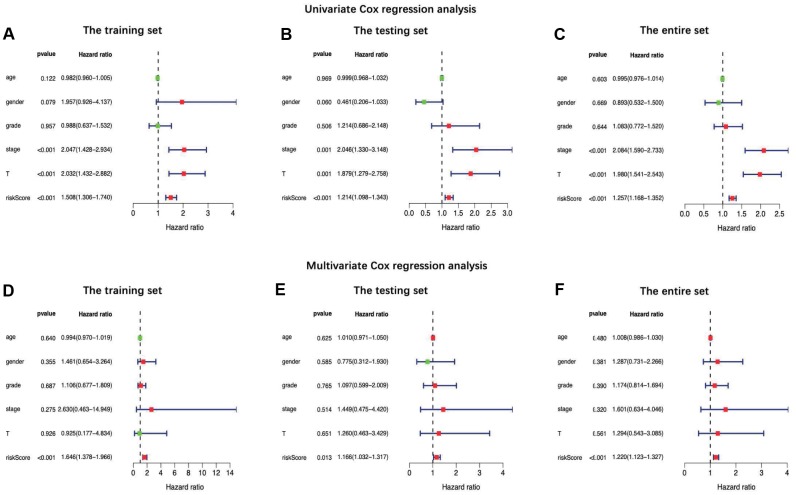
Univariate (**A–C**) and multiple (**D**–**F**) regression analysis of hepatocellular carcinoma and the relationships between the age, gender, grade, stage, T stage, distant metastasis, lymph node metastasis and riskScore in the training set (**A** and **D**), the testing set (**B** and **E**) and the entire set (**C** and **F**). Green dot means hazard ratio (HR) median value is less than 1, red dot means HR median value is greater than 1.

**Figure 6:**
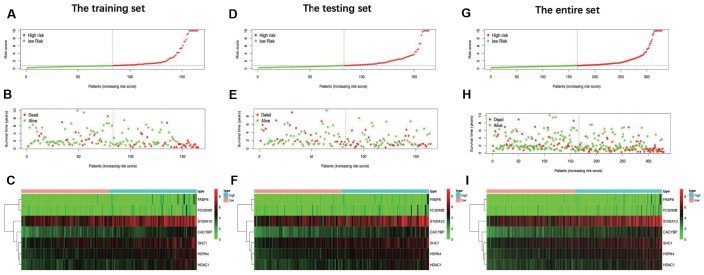
Distribution of risk score, overall survival (OS), gene expression in (**A**–**C**) training set, (**D**–**F**) testing set and (**G**–**I**) entire set. Distribution of risk score, OS and heat map of the expression of 7 signature genes in low-risk and high-risk groups are listed in the picture from top to bottom.

The prognostic signature was established based on the LASSO-penalized Cox regression analysis results (Figure 3A and 3B), so as to stratify TCGA-LIHC patients as two groups, namely, the low-risk group and the high-risk group, with discrete clinical outcomes with regard to OS. To be specific, the following formula was adopted for calculation: [Expression level of HSPA4* (0.0361)] + [Expression level of S100A10* (-0.0043)] + [Expression level of CACYBP* (0.0416)] + [Expression level of FABP6 * (0.0797)] + [Expression level of HDAC1* (0.0247)] + [Expression level of FCGR2B* (0.1551) + [Expression level of SHC1* (0.0091)] (Table 1). Figure 3C shows the comparisons of survival differences between the two groups in training set (P < 0.001). Moreover, such findings were further verified in the testing set and the entire set (P < 0.01) (Figure 3D and 3E). Additionally, the AUC for 1-year OS were 0.821, 0.833 and 0.828 in the training set, the testing set and the entire set, separately, which indicated moderate potentials for the metabolic gene signature to monitor survival (Figure 3F–3H). Our model achieved the greatest AUC value compared with those of other clinicopathological characteristics, which also reflected its excellent predicting ability. In the entire set, the low-risk group showed markedly superior prognosis to the high-risk group among all (≤65/>65; Figure 4A and 4B), sex (female/male; Figure 4C and 4D), stage (I+II/III+IV; Figure 4E and 4F), grade (G1+G2/G3+G4; Figure 4G and 4H) and T stage (T1+T2/T3+T4; Figure 4I and 4J) subgroups. Similar results were also observed in patients without distant metastasis (Figure 4K) and lymph node metastasis (Figure 4L). Additionally, the multivariate analysis revealed that the as-constructed immune gene signature could become an independent predictor after other clinicopathological parameters were adjusted (Figure 5). Figure 6 depicts the risk score distribution (A, D, G), survival status (B, E, H) and expression of the metabolic gene signature (C, F, I) in the training set (A–C), the testing set (D–F) and the entire set (G–I). Clearly, their distributions were similar, thus supporting the robust predictive capacity of our immune related gene-based risk score assessment model. Owing to the significant clinical value of the signature genes, we embarked on a comprehensive investigation of their molecular characteristics. Genetic alternations of these genes in LIHC (TCGA-PanCancer Altas) were explored via cBioPortal, and amplification was found to be the most commonly occurring type of mutation (Supplementary Figure 1). Besides, there were 4 genes (*SHC1*, *FCGR2B*, *S100A10* and *CACYBP*) with a mutation rate ≥ 5%.

**Table 1:** Seven immune-related signature genes identified from Cox regression analysis from TCGA.

**id**	**coef**	**HR**	**HR.95L**	**HR.95H**	**P-value**
HSPA4	0.03606573	1.03672399	1.00620270	1.06817108	0.01800376
S100A10	0.00428466	1.00429385	1.00126694	1.00732992	0.00540109
FABP6	0.07972664	1.08299099	1.01358048	1.15715476	0.01831907
CACYBP	0.04157927	1.04245579	0.99974327	1.08699314	0.05142275
HDAC1	0.02472582	1.02503404	1.00243637	1.04814112	0.02971183
FCGR2B	0.15512764	1.16780702	1.07395448	1.26986129	0.00028443
SHC1	0.00914823	1.00919021	1.00025144	1.01820885	0.04386807

Coef, coefficient; HR, hazard ratio; L, low; H, high.

### PCA proved the model grouping capacity

**Figure 7:**
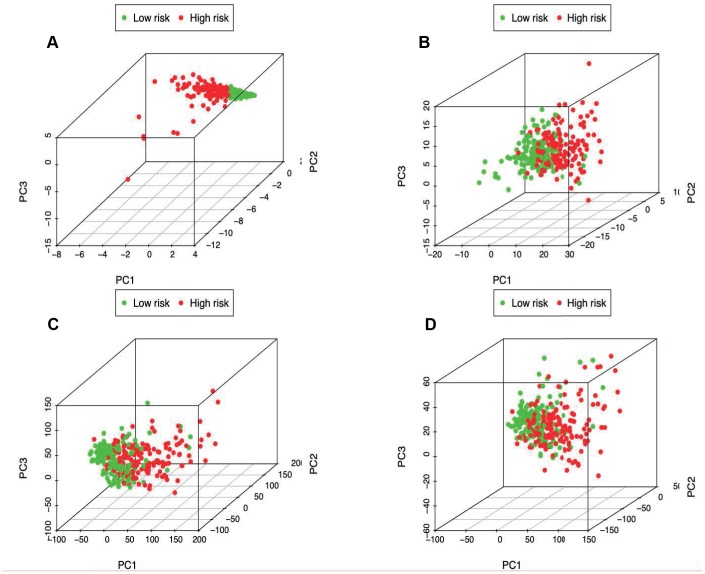
Principal components analysis between low- and high-risk groups based on (**A**) immune-related signature, (**B**) immune-related genes, (**C**) differently expressed genes and (**D**) the entire gene expression profiles.

PCA was further conducted to examine the difference between low- and high-risk groups based on the immune-related signature (Figure 7A), immune genes (Figure 7B), differently expressed genes (Figure 7C) and the entire gene expression profiles (Figure 7D). The results obtained based on our model showed that low- and high-risk groups were generally distributed at different directions. Nonetheless, the distributions of high- and low-risk groups displayed in Figure 7B–7D were relatively scattered, which confirmed that our prognosis signature was capable of distinguishing the high-risk group from the low-risk group.

### Correlation of the prognostic model with clinicopathological characteristics

**Figure 8:**
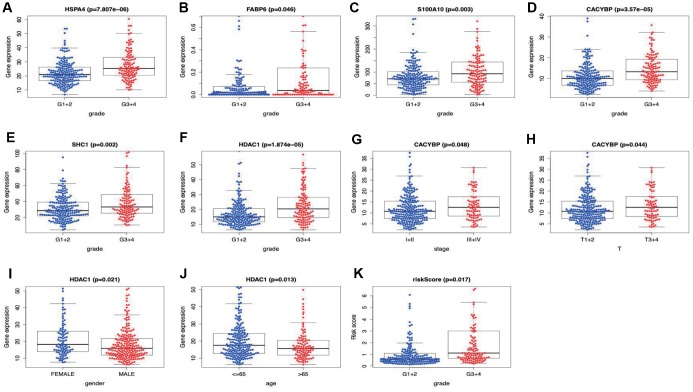
**Correlation of the prognostic immune-relate signature with clinicopathological characteristics.** *HSPA4*, *FABP6, S100A10, CACYBP, SHC1* and *HDAC1* were associated with a higher tumor grade (**A**–**F**), *CACYBP* was linked with a higher clinical stage (**G**) as well as T stage (**H**). The expression level of *HDAC1* was significantly enhanced in female patients (**I**) and patients younger than 65 years old (**J**). Risk score derived from our model was significantly associated with higher tumor grade (**K**).

Two hundred and twenty-one patients with complete information including gender, age, tumor grade, clinical stage, T stage, lymph node metastasis and distant metastasis were included in TCGA-LIHC cohort. Among the signature genes we researched, *HSPA4*, *FABP6, S100A10, CACYBP, SHC1* and *HDAC1* were associated with a higher tumor grade (Figure 8A–8F), *CACYBP* was linked with a higher clinical stage as well as T stage (Figure 8G and 8H). Additionally, the expression level of *HDAC1* was significantly enhanced in female patients and patients younger than 65 years old (Figure 8I and 8J). Afterwards, risk score derived from our model was significantly associated with higher tumor grade (Figure 8K).

### Functional enrichment analysis revealed different states between high- and low-risk groups

**Figure 9:**
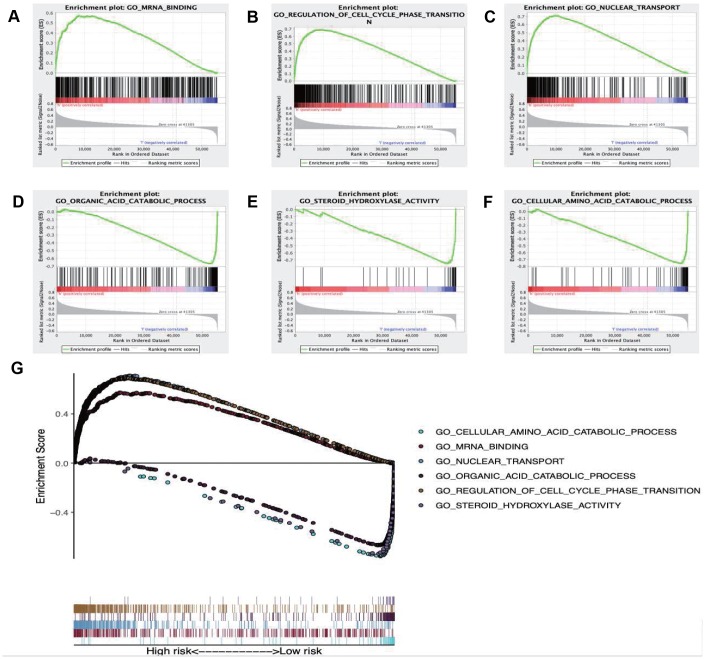
Enrichment plots of Gene Ontology annotation from gene set enrichment analysis (GSEA). GSEA results showing (**A**) mRNA binding, (**B**) Regulation of cell cycle phase transition, (**C**) Nuclear transport were differentially enriched in high risk phenotype, while (**D**) Organic acid catabolic process, (**E**) Steroid hydroxylase activity (**F**) Cellular amino acid catabolic process were closely correlated with the low risk phenotype. (**G**) Summarizes the above six gene sets.

**Figure 10:**
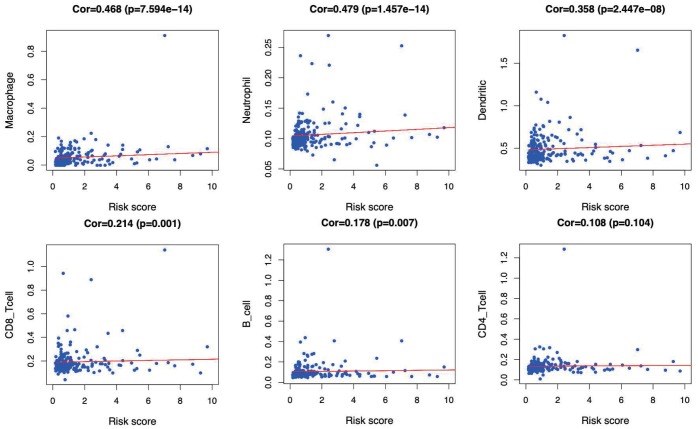
**Relationships between the immune-related prognostic index and infiltration abundances of six types of immune cells.** The correlation was performed by using Pearson correlation analysis. (**A**) macrophages; (**B**) neutrophils; (**C**) dendritic cells; (**D**) CD8^+^T cells; (**E**) B cells; and (**F**) CD4^+^T cells.

GSEA was performed to further investigate the differences between the high- and low-risk groups. The results revealed that the GO molecular function “mRNA binding” (Figure 9A), biological process “Regulation of cell cycle phase transition” (Figure 9B) and “Nuclear transport” (Figure 9C) were differentially enriched in high-risk phenotype (P< 0.01), while biological process “Organic acid catabolic process” (Figure 9D), molecular function “Steroid hydroxylase activity” (Figure 9E) and biological process “Cellular amino acid catabolic process” (Figure 9F) were closely correlated with the low risk phenotype (P< 0.01). In addition, KEGG pathway analysis suggested that the genes in high-risk group were mainly enriched in the “Spliceosome” (Supplementary Figure 2A), “RNA degradation” (Supplementary Figure 2B) and “Oocyte meiosis” (Supplementary Figure 2C) (P< 0.01); in addition, the “Complement and coagulation cascades” (Supplementary Figure 2D), “Glycine serine and threonine metabolism” (Supplementary Figure 2E) and “Primary bile acid biosynthesis” (Supplementary Figure 2F) were primarily enriched in low risk group (P< 0.01). Moreover, the immune status between the low- and high- risk group was also examined via GSEA, and the results suggested that the differentially expressed genes between these two groups were enriched in the immunological signature gene sets (c7. All. V7.0. symbol). According to the normalized enrichment score (NES), the top six immune related gene sets are shown in Table 2. Furthermore, the relationship of the prognostic signature with immune cell infiltration in TCGA-LIHC patients was investigated to examine whether the risk score partially reflected the tumor immune microenvironment status (Figure 10). Our results suggested that, for high risk patients in the entire set, the levels of macrophages (Cor=0.468; p=7.594e−14), neutrophils (Cor=0.479; p=1.475e-14) and DCs (Cor=0.358; p=2.447e−08), significantly increased in tumor microenvironment (TME) (Figure 10A–10C). Besides, CD8^+^ T cells (Cor=0.214; p=0.001) (Figure 10D) and B cells (Cor=0.178; p=0.007) (Figure 10E) were also showed association with high-risk group.

**Table 2:** Immune-related gene sets that associated with high-risk group.

**NAME**	**ES**	**NES**	**NOM p-val**	**FDR q-val**
HEALTHY VS HIV AND SIV INFECTED DC UP	0.6998564	2.2639873	0	0
CTRL VS TIV FLU VACCINE PBMC 2008 DN	0.6825686	2.2599075	0	0
NAÏVE VS GC B CELL DN	0.6806816	2.2485397	0	0
CTRL VS POLYIC STIM DC 3H UP	0.6934012	2.2360585	0	0
NAÏVE CD4 TCELL VS DAY5 IL4 CONV TREG DN	0.713608	2.2357495	0	0

ES, enrichment score; NES, normalized enrichment score; NOM, nominal; FDR, false discovery rate.

## DISCUSSION

Studies on the revealing of new biomarkers for diagnosis and treatment of LIHC is in full swing [15, 16], and the relationship between immunity and LIHC has always been a research hotspot. Recently, Moeini et al*.* identified an immune-related gene expression pattern in liver tissues of patients with early-stage LIHC, which is associated with risk of LIHC progression in patients with cirrhosis [17]. The researchers also revealed that administration of nintedanib or aspirin and clopidogrel to mice with chronic liver inflammation caused loss of the above-mentioned gene expression pattern and development of fewer and smaller liver tumors, which further demonstrated the utility and usability of the immune signature. Although the significance of IRGs in LIHC progression and immunotherapy has been explored, there is still an urgent need for comprehensive genome-wide profiling studies to explore their clinical significance and underlying molecular mechanisms. The current study investigated the value of IRGs in LIHC in a holistic and comprehensive way, and our results further revealed their clinical significance and potential molecular characteristics. As suggested by our results, the identified IRGs were tightly involved in LIHC initiation and progression, which might also predict the prognosis for LIHC patients. As a result, these genes might give scope to their potentials as the valuable clinical biomarkers. Also, a regulatory network was constructed in this study, which added several novel TFs and shed more light on the interactions between factors. Furthermore, an individualized immune prognostic model was proposed based on the selected differentially expressed IRGs, so as to measure immunocyte infiltration and to assess the potential clinical outcomes.

As an inflammation-associated tumor, the immune microenvironment of LIHC is well evidenced to shape tumor progression, invasion and metastasis through establishing a symbiotic relationship with cancer cells [8]. Researchers have gained an insight into the tumor-linked infiltrated inflammatory environments, nonetheless, numerous aspects of LIHC immune-associated molecular mechanisms remain unclear, and the biomarkers for predicting the treatment response are still lacking. A body of studies have uncovered the DEGs between LIHC and non-tumor samples [18–20], which contributes to the fundamental understanding towards the pathogenesis of LIHC at genetic level. For example, Carone et al. detected the expression levels of 579 immune response-related genes in 30 frozen LIHC liver tissue samples and 33 normal tissues, and demonstrated that the longer time to LIHC recurrence (TTR) was associated with up-regulation of immune response- and inflammation-related genes in tumor tissues, whereas down-regulation of these genes in normal tissues [21]. In our study, the immunocyte abundances that served as a means to characterize the immune microenvironment of LIHC, was used in combination with the immuno-genomic profiles and the corresponding clinical significance, which helped to explore the immune landscape from a novel perspective.

The acquisition of the invasive traits in cancer cells depends on a succession of genomic alterations. Therefore, the current investigation was started from the differences in the expression profiles of immune genes between LIHC and adjacent normal liver tissues, so as to uncover the relationships between these profiles and the immune microenvironment, and to illustrate the potential clinical implications. After identifying the prognosis-related immune genes, the TF-mediated network was constructed, for the sake of exposing the vital TFs that regulated the identified IRGs and revealing the underlying molecular mechanisms. Among them, chromobox protein homolog 3 (CBX3), which has the highest number of nodes related to survival-related immune genes, has been illustrated to promote tumor proliferation and predict poor survival in LIHC [22]. Besides, host cell factor C1 (HCFC1), a chromatin-associated transcriptional regulator that may also play an overall regulatory role in survival related immune genes, exerts its function in the cell division cycle during cell culture, embryogenesis as well as adult tissue [23, 24]. In addition, HCFC1 is also proven to play a significant role in liver regeneration and show moderate co-expression with ARIDIA. ARIDIA encodes the BAF250a subunit of the SWI/SNF complex, and it plays an opposite and complex regulatory role at various stages of LIHC initiation and development [25]. Motallebipour et al. demonstrated that HCFC1 possessed the binding sites with the sterol response element-binding protein-1 (SREBP-1), which might be involved in the lipid metabolic reprogramming in LIHC [26]. Noteworthily, both the SWI/SNF chromatin remodeling complex and the aberrant lipid metabolism are demonstrated to be associated with the immune microenvironment of LIHC, implying a potential genetic link among these three. Previous studies have also shown that, the HCFC1 expression level in mutant β-catenin LIHC is lower than that in non-mutant β-catenin LIHC [27]. Additionally, LMNB1, another TF revealed by our regulatory network, is shown to be markedly up-regulated in LIHC, which is distributed in patient plasma [28]. On the other hand, LMNB1 functions in the nuclear envelope lamina, possesses the transcriptional coregulatory activity, and has an important role in DNA replication, cell aging, and stress responses. Besides, it is positively correlated with tumor stage, tumor size, and number of nodules, therefore rendering itself a biomarker for early LIHC in tumor tissues and plasma [29]. In addition, SMARCA4 may enhance the growth and invasion of LIHC cells [30], while NRF1 is involved in stimulating the mitochondrial DNA (mtDNA) replication and transcription within LIHC [31], all of which are revealed in the network. Taken together, our results demonstrate a relatively comprehensive immune gene regulatory network associated with LIHC, yet further in-depth studies are warranted to examine the interactions between immune genes and the effects on liver cancer cells.

In this study, an immune-based prognostic signature was established to develop a simple and convenient protocol for observing the immune status and predicting the clinical outcomes for LIHC patients. This prognostic signature was based on 7 differentially expressed survival related IRGs in LIHC, which had favorable clinical viability. For the training set, LIHC patients in low-risk group had longer OS than those in high-risk group (P=3.309e−05), and similar results were also obtained in the testing set (P=4.231e−03) and the entire set (P=8.618e−07). Furthermore, our data showed that the prognosis signature performed moderately in prognosis prediction. In terms of the clinical utility, there was a significant correlation between the prognostic signature and tumor grade (P=0.017), which meant that the risk score calculated by the model we built was significantly higher in advanced grade cases. Besides, the as-constructed prognosis model also displayed the potentials to predict the differential prognosis between high- and low-risk groups, when patients were stratified by age (≤65/>65), clinical stage (I and II/III and IV), grade (G1 and G2/G3 and G4) and T stage (T1 and T2/T3 and T4). It implies that under diverse clinicopathological conditions, our signature is efficacious for predicting the prognosis of patients. Further PCA confirmed that our prognosis signature had sound grouping capacity. Additionally, the as-constructed signature was compared with the other two prognostic immune signatures previously published by Chew et al. to the TCGA dataset, and both of them had reached a lower significance level, with the P-values of 0.0092 and 0.0067, respectively [32, 33]. The model structured in this study exerted its own strengths in initially determining the patient prognosis and rapidly adjusting the treatment plans based on the expression of immune genes and the immunocyte infiltration levels.

Moreover, the GO item “mRNA binding” and the KEGG pathway “Spliceosome” were proved to be most significantly associated with the high-risk group. Some molecules involved in mRNA binding, such as insulin-like growth factor II mRNA-binding protein 3 (IMP3), has been demonstrated to promote tumor invasion and predicts early recurrence and poor prognosis in LIHC [34]. With regard to the latter, previous literature elaborated that genes in spliceosome pathway, which were shown to be upregulated in tumor tissue compared with normal liver tissue, played a role in progression of LIHC [35, 36]. The two aspects mentioned above might have a bearing on the poor prognosis of high-risk group. GSEA also revealed different immune states in high-risk group compared with low-risk group. Moreover, the relationships between the immune-related prognostic index and immunocyte infiltration were deliberated to reflect the immune microenvironment status of LIHC. Of interest, our analysis indicated that the signature showed positive correlation with the infiltration of 5 kinds of immune cells, especially macrophages, neutrophils and DCs, indicating that the higher infiltration levels of these cells might be observed in the high-risk patients. Consistently, recent studies have expounded that the high densities of tumor-infiltrating macrophages and neutrophils predict the poor prognosis for primary LIHC patients. Intramural neutrophil infiltration is elaborated to be promoted by the CXCL5 and CXCR2-CXCL1 axis; besides, it is remarkably related to the shorter OS and LIHC recurrence, and is also taken as an independent prognostic factor [37, 38]. Therefore, patients with high risk scores that derived from our signature may be candidates for neutrophil targeted therapies. He et al*.* also illustrated that the co-inhibitory molecule programmed cell death ligand 1 (PD-L1) was observed to be overexpressed on infiltrating neutrophils from patients with LIHC, and further pointed out that the PD-L1+ neutrophils from patients with LIHC effectively suppressed the proliferation and activation of T cells, which could be partially reversed by the blockade of PD-L1 [39]. Thus, we speculated that the high-risk patients in our research may benefit from anti-PD-L1 antibodies. Furthermore, the increased infiltration of tumor-associated macrophages (TAM) (dominated by the M2 macrophages), which may be due to the deletion of the Hippo signaling, has been reported to produce the Wnt/β-catenin signaling and trigger the elevated intramural FoxP3+ Treg population, while this in turn accelerates LIHC progression [40–42]. Moreover, Li et al*.* have elaborated that therapeutic blocking of the CCL2/CCR2 axis inhibits the recruitment of inflammatory monocytes, infiltration and M2-polarisation of TAMs, resulting in reversal of the immunosuppression status of the LIHC microenvironment and activation of an anti-tumorous CD8+ T cell response [43], which may be applicable to high-risk patients in our study. In line with this, Chen et al*.* demonstrated that upregulation of B7-H1 expression is associated with macrophage in LIHC, and anti-inflammatory therapies targeting on TAM or signaling pathways like NF-kB and STAT3 may downregulate the B7-H1 expression on malignant cells and enhance the efficacy of immunotherapy based on tumor-specific CD8+ T cells [44]. In addition, accumulating evidence reveals a role of DCs as an adverse prognostic factor for LIHC. For instance, Zhou et al. set forth that, the intra-tumoral infiltration by plasmacytoid DCs was a novel indicator of the poor prognosis for LIHC patients, which might be achieved through inducing the immune tolerogenic and inflammatory TME comprising regulatory T and IL-17-producing cells [45]. Such results have underscored the importance of tumor-associated DC cells in predicting the prognosis for LIHC patients. Our results confirmed that immunocytes were essential for LIHC progression, and suggested that the signature we constructed might potentially serve as the predictor for elevated immunocyte infiltration, which coincided with previous reports. Additionally, the immune related signature may potentially provide an instruction for treatment of LIHC. Nevertheless, the immune microenvironment of LIHC is intricate, and the role of immunocytes in LIHC has not been fully illustrated yet, which requires more efforts.

Nonetheless, some limitations should be noted in this study, which should be taken into consideration when interpreting our results. Firstly, it remained unclear about whether pretreatment in LIHC patients, like hepatic resection or transarterial chemoembolization, affected the immune contexture composition, due to the insufficient detailed clinical information. Secondly, transcriptomic analysis only reflected some aspects of the immune status, rather than the global alterations. Thirdly, the immunocyte-specific gene sets applied in this study were limited to 6 major immunocyte types, so the differences in more specialized immunocyte subtypes (like the differently polarized macrophages or myeloid-derived suppressor cells) might not be recognized in this study, while they were known to be mechanistically linked to LIHC progression and stage [46, 47]. Finally, our results were not validated via another independent cohort, which was also a limitation of this study, and the reliability of our molecular results was still challenged by the lack of experiments in vitro or in vivo.

## CONCLUSIONS

To sum up, this study projects the relevance of immune microenvironment for LIHC outcomes and proposes a concise signature for the relationship between immune status and LIHC patient prognosis. The prognostic signature established in this study may be of great clinical significance, and this study can provide new insights in developing new immunotherapies for LIHC. The bioinformatic approach exposed in this study also embodies a straightforward methodology to construct other human malignancies, which will make a crucial difference to guide future clinical studies.

## MATERIALS AND METHODS

### Clinical samples and data acquisition

The transcriptomic RNA-sequencing data of LIHC samples were downloaded from the TCGA data portal (https://cancergenome.nih.gov/). Meanwhile, the clinical data of these patients were also downloaded and extracted. Furthermore, the missing follow-up information was filtered out, and 329 unique LIHC samples, which were randomized as the training group (n=165) and the testing group (n=164) using the R package “caret” [48], were incorporated into subsequent analysis. Of them, the training set was adopted to establish a prognostic immune gene signature, whereas the testing set and the entire set were employed to validate the predictive power of the as-established signature.

A list of IRGs was obtained from the Immunology Database and the Analysis Portal (ImmPort) database [49]. Typically, ImmPort is a database that accurately and timely updates the immunology data, and data shared through ImmPort lay down a powerful foundation for immunologic research. More importantly, this database provides a list of IRGs for cancer research, and these genes are identified to actively participate in the process of immune activity.

### Analysis of differentially expressed genes (DEGs)

To select IRGs that were associated with LIHC, the differentially expressed IRGs between LIHC and adjacent non-tumor samples were screened using the limma package of R software (http://bioconductor.org/packages/limma/). Afterwards, differential gene analysis was conducted among all transcriptional data, with the false discovery rate (FDR) of < 0.05 and the log2 |fold change| of > 1 as the cutoff values. Then, the differentially expressed IRGs were extracted from all DEGs. Subsequently, the Gene Ontology (GO) annotation and Kyoto Encyclopedia of Genes and Genomes (KEGG) pathway functional enrichment analysis were carried out to probe into the potential molecular mechanisms of the differentially expressed IRGs.

### TFs extraction and regulatory network construction

Clinical data downloaded from the TCGA data portal were collected to extract the overall survival time. Later, the survival related IRGs were selected upon univariate COX analysis carried out using the survival package of R software. To explore the interactions between these genes, a regulatory network was constructed. TFs are the important molecules that directly control the gene expression level. The Cistrome Cancer is a data source that integrates the cancer genomic data from TCGA with over twenty-three thousand ChIP-seq and chromatin accessibility profiles, which can provide the regulatory links between TFs and transcriptomes [50]. Generally, TFs are compared with differential genes of all transcriptional data, so as to identify the differentially expressed TFs and to draw the heatmap and volcano map. Besides, the differential TFs are also connected with the survival-related immune genes and the mapping regulatory network using the Cytoscape software version 3.7.2 [51].

### Development and verification of the IRG-based prognostic signature

Through univariate Cox regression analysis and least absolute shrinkage and selection operator (LASSO)- penalized Cox regression analysis [52], we develop the IRG-based prognostic signature. Notably, LASSO is the penalized regression, which employs an L1 penalty to shrink the regression coefficients toward zero, thereby eliminating numerous variables based on the principle that fewer predictors are selected in the presence of a larger penalty. Thereafter, an IRG-related prognostic signature was built to predict patient overall survival (OS). The R package “survival” and “survminer” was used to explore the optimal cut-off of risk score and drawn the Kaplan–Meier survival curve. The R package “survivalROC” [53] was used to investigate the time-dependent prognostic value of the gene signature [54]. A two-sided log-rank P < 0.05 were considered significant for survival analysis. Afterwards, both univariate and multivariate Cox regression analyses were performed to ascertain whether the signature predicted prognosis independently from the conventional clinical factors (such as age, sex, grade, clinical stage and TNM stage). Besides, the correlation of the signature with the clinicopathological characteristics was also analyzed through the R package “ggpubr” [55]. Also, principal components analysis (PCA) was also conducted as the dimension-reducing procedure to identify a small set of synthetic variables, so as to explore the model grouping capacity. Notably, PCA is a statistical technique to determine the key variables in a multidimensional data set, which explains the observational differences and is utilized to simplify the analysis and visualization of the multidimensional data sets [56]. PCA was implemented through the limma [57] and scatterplot3d [58] packages.

### Gene set enrichment analysis (GSEA)

GSEA is a computational approach to determine whether a priori defined gene set shows statistically significant and concordant differences between two biological states [59]. To reveal potential underlying Kyoto Encyclopedia of Genes and Genomes (KEGG) pathways of the gene signature, GESA was performed in this study using the JAVA program (https://www.broadinstitute.org/gsea), so as to identify enriched terms in TCGA-LIHC cohort. P < 0.05 and a false discovery rate q < 0.25 were considered statistically significant. Following 1000 permutations, the top 3 pathways in terms of the normalized enrichment score (NES) in each group were employed in multiple GSEA, so as to demonstrate a whole picture of the signaling pathways involved in the metabolic signature in LIHC.

### Relationships of immune-related prognostic index with immune cell infiltration

The TIMER online database, which is a web resource to systemically evaluate the clinical impact of various immune cells on diverse cancer types, analyzes and visualizes the abundances of tumor-infiltrating immune cells [60]. It covers 10,009 samples across 23 cancer types from TCGA to estimate the abundance of six tumor-infiltrating immune cell subtypes, including B cells, CD4 T cells, CD8 T cells, macrophages, neutrophils, and dendritic cells (DCs). Therefore, it can be easily used to determine the relationship of immune cells infiltration with other parameters. In this study, the immune infiltrate levels of TCGA-LIHC patients were downloaded, and the associations of the prognostic signature with immune cells infiltration were calculated.

### Statistical analysis

The R (v.3.6.1) software was employed for all statistical analyses. Pearson χ2 test or Fisher’s exact test were used to explore qualitative variables as appropriate. If not specified above, P < 0.05 was considered statistically significant.

## Supplementary Material

Supplementary Figures

Supplementary Table 1
