# Supplementary material for: Development of an immune-related prognostic index associated with hepatocellular carcinoma
Source: Aging (Albany NY). 2020 Mar 19;12(6):5010–30. doi: 10.18632/aging.102926 (PMC7138589; doi:10.18632/aging.102926)
Supplement: Supplementary Figures [file aging-12-102926-s002..pdf]

SUPPLEMENTARY FIGURES

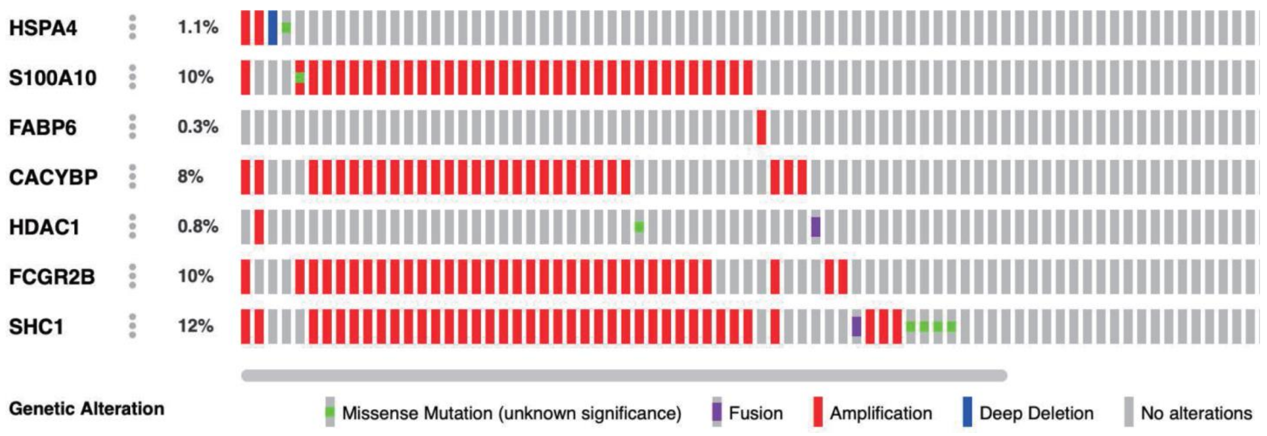

Supplementary Figure 1. Genetic alternations of signature genes in TCGA-LIHC. *SHC1* is the gene with highest mutation frequency. There are 4 genes with a mutation rate ≥5%.

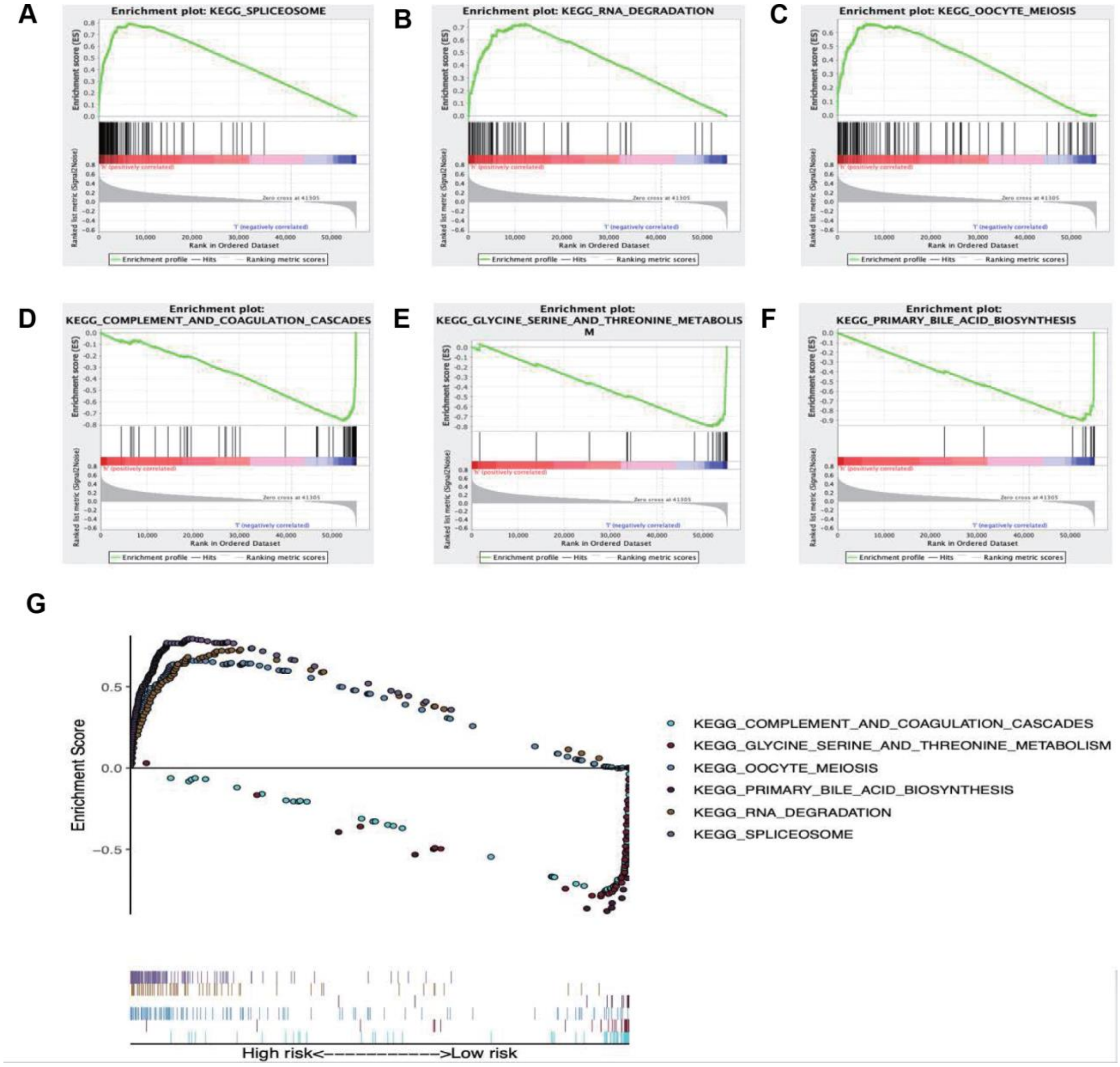

Supplementary Figure 2. Enrichment plots of Kyoto Encyclopedia of Genes and Genomes analysis from gene set enrichment analysis (GSEA). GSEA results showing (**A**) Spliceosome, (**B**) RNA degradation, (**C**) Oocyte meiosis were differentially enriched in high risk group, while (**D**) Complement and coagulation cascades, (**E**) Glycine serine and threonine metabolism, (**F**) Primary bile acid biosynthesis were primarily enriched in low risk group. (**G**) Summarizes the above six gene sets.
