# Supplementary material for: Development of an immune-related prognostic index associated with hepatocellular carcinoma
Source: Aging (Albany NY). 2020 Mar 19;12(6):5010–30. doi: 10.18632/aging.102926 (PMC7138589; doi:10.18632/aging.102926)
Supplement: Supplementary Table 1 [file aging-12-102926-s001..pdf]

SUPPLEMENTARY TABLE

Supplementary Table 1. General characteristics of LIHC-specific survival-related immune genes.

id	HR	HR.95L	HR.95H	P-value
CACYBP	1.0917619	1.05795119	1.12665315	4.51E-08
HDAC1	1.05022551	1.03097483	1.06983565	2.08E-07
PSMD14	1.10778447	1.06262069	1.15486781	1.44E-06
HSPA4	1.05964018	1.03404643	1.08586739	3.42E-06
NRAS	1.08973904	1.05091756	1.12999461	3.43E-06
ISG20L2	1.16874	1.09328287	1.24940509	4.67E-06
TNFRSF11A	2.07200697	1.51253076	2.83843013	5.71E-06
IFI30	3.17933487	1.90457983	5.30729671	9.68E-06
S100A10	1.00566657	1.00308672	1.00825306	1.62E-05
PPIA	1.01599581	1.00862786	1.02341759	1.93E-05
FABP6	1.12300205	1.06476789	1.18442114	1.96E-05
FABP5	1.07739142	1.04069686	1.11537981	2.48E-05
SHC1	1.01663274	1.00869591	1.02463202	3.70E-05
IL2RG	1.02199182	1.01147917	1.03261373	3.73E-05
IKBKE	1.27102829	1.12666386	1.43389077	9.67E-05
HGF	1.1241683	1.05920587	1.19311495	0.00011624
PSMD10	1.0656461	1.0311687	1.10127627	0.00015121
BIRC5	1.0404312	1.01860922	1.06272068	0.0002475
CCL8	1.19891453	1.08519549	1.32455034	0.00035977
GBP2	1.03201284	1.01428214	1.0500535	0.00036553
PDGFRL	1.21816123	1.0896081	1.36188119	0.00052404
IL17D	1.09463878	1.03846349	1.15385284	0.00076792
SPP1	1.00060692	1.00024964	1.00096432	0.00086893
BRD8	1.20845349	1.08066447	1.35135362	0.00089898
TAP2	1.08531931	1.03409177	1.13908459	0.00090377
TAP1	1.01069017	1.00430185	1.01711913	0.00101324
S100A11	1.00167522	1.00067095	1.00268051	0.00107363
PSME3	1.05152035	1.02029916	1.08369692	0.00108801
DCK	1.18182999	1.06736158	1.30857448	0.0013083
ZYX	1.01343573	1.0050076	1.02193455	0.00173453
CCL20	1.00559114	1.00206949	1.00912517	0.00183976
NDRG1	1.00647063	1.00237442	1.01058359	0.00193684
SEMA5B	1.26004029	1.08844097	1.45869329	0.00197114
EED	1.52350585	1.16595225	1.99070766	0.00203529
PSMD2	1.02110969	1.00726704	1.03514258	0.00270242
RFX5	1.07783148	1.0263163	1.13193243	0.00270413
PLXNA2	1.33484634	1.10350362	1.61468864	0.00293744
NFKBIE	1.04940072	1.01632056	1.08355761	0.0031719

CDK4	1.03302289	1.01084017	1.0556924	0.00335215
LCNL1	2.06794791	1.26218984	3.38808668	0.00392231
IL27RA	1.15188728	1.04616157	1.2682977	0.00399336
FGF13	2.65086219	1.36077852	5.16400742	0.00416462
PML	1.18079808	1.05286741	1.32427322	0.00450452
PLXNA1	1.18084436	1.0519579	1.32552205	0.00481816
FCGR2B	1.11560295	1.03356267	1.20415528	0.00500003
IRF5	1.18229283	1.05148895	1.32936854	0.00512223
PPARG	1.10740754	1.03047593	1.19008259	0.00548345
FPR1	1.12723089	1.03554744	1.22703165	0.00565789
CCL14	0.35186891	0.16776124	0.73802346	0.00571367
S100A6	1.00164935	1.00047594	1.00282414	0.00585868
KITLG	1.27115114	1.07133671	1.50823286	0.00596518
HLA-DOB	1.06529841	1.01805969	1.11472904	0.00626831
MAPK3	1.08276978	1.02220825	1.14691934	0.00677033
IDO1	1.07592028	1.0200119	1.13489308	0.00719388
CSPG5	1.67901922	1.14889605	2.45375164	0.00742866
NR6A1	1.37807871	1.08943246	1.74320208	0.00748934
WNT5A	1.19136252	1.04748929	1.35499683	0.00766411
TRAF3	1.28965446	1.06884011	1.55608741	0.0079365
PLAU	1.04381479	1.01096212	1.07773506	0.00858497
MICB	1.15494195	1.03698719	1.28631376	0.00877407
LPA	0.84675749	0.74669709	0.96022638	0.00952743
